# Supplementary material for: Patterns of Intron Gain and Loss in Fungi
Source: PLoS Biol. 2004 Nov 30;2(12):e422. doi: 10.1371/journal.pbio.0020422 (PMC532390; doi:10.1371/journal.pbio.0020422)
Supplement: Table S1 — Also available at http://genes.mit.edu/NielsenEtAl/. (4.3 MB ZIP). [file pbio.0020422.st001.zip › NielsenEtAl/html/719.html]

AN2077.1.NCU03961.1.MG06500.1.FG08439.1


```
 CLUSTAL W (1.82) Multiple Sequence Alignments - Introns Inserted


Sequence 1: MG06500.1	636 aa
Sequence 2: FG08439.1	687 aa
Sequence 3: NCU03961.1	645 aa
Sequence 4: AN2077.1	728 aa
Alignment Length: 766 aa
Number Identitical Residues: 281 aa
Alignment Score (without introns) 14822


MG06500.1 	-----~----~---------------------------MKLQNSGTIPV~YTVSGASTAR
NCU03961.1	-----~----~---------------------------MKLTNPGAVPV2YTVSGPSTAR
FG08439.1 	MGRQK0IGAV1ASAPTQNLLPTVEQQPLFLRLTKNTVAMKLSNPGTVPV~YTISGASSAR
AN2077.1  	-----~----~---------------------------MKLSNQSEVPV~YTISGSNTAR
          	                                      *** * . :** **:**..:**

MG06500.1 	SLPEWLSRKKKQSLKNDPEYANRIELLQDFEFEEASMCVRVSEDGDWAMSTG~TYKPQIH
NCU03961.1	PLPEWLARRRKRSAKYEPEELNNFELLQNFDFEEASNCVRVSEDGNWIMSSG~TYKPQFH
FG08439.1 	PLPDWLARRRKRSLKDDAEYQNRVELLQDFEFEEASNCIRVSEDGGWIMSTG~TYKPQIH
AN2077.1  	PLPEWLARRRKRSLKDDPEYANRIELLQDFEFEEASQCIRVSEDGDWVMSTG1TYKPQIH
          	.**:**:*::*:* * :.*  *..****:*:***** *:******.* **:* *****:*

MG06500.1 	THHLSSLSMSYARHTSSLNQTFVLLSSDVSKSLHLQNDRRLELHTLAGCHYHVRLPRFGR
NCU03961.1	VHNTQELSLSFARHTKSENTTFIMLSQDYTKSVHLQSDRSIEFHTPMGCHYEVRLPRFGR
FG08439.1 	VHNLSQLSLSFARHTNALNHSFELLSSDYSKSIHLQTDRRLEFHTPMGCHYEVRLPRYGR
AN2077.1  	THHLPQLSLSWARHTDALNTTFQLLSSDYSKSIHLQSDRSLEFHTPGGCHYRTRLPRYGR
          	.*:  .**:*:****.: * :* :**.* :**:***.** :*:**  ****..****:**

MG06500.1 	DLAYDRHSTEAFVPATGLDSDGK--AEVFRLNLELGRFMKSYQIDCGTDDGVEQG---LQ
NCU03961.1	DLAYLRQSTEVLIPSVGLSADGSGMGEVFRLDLERGQFLRPWQVDVGEDE--PGAG--LQ
FG08439.1 	ALVYDRQSTEALIPSVGLDGDGK--GEVFRMNLELGRFMKSYQVDVGGDDELTGGG--LQ
AN2077.1  	DLIYDRASTEALVPAVGVNADGM--GEVFRLNLELGRYMRSFEVDVGGDDFTSAGGGALQ
          	 * * * ***.::*:.*:..**   .****::** *::::.:::* * *:    ...:**

MG06500.1 	GSINVGSVNTVALAENTHNLSAFGTSIGTVEFWDPRTKSRVATIG-----GHD---GEVT
NCU03961.1	GSINVGAVNVAAVAESTHGLCAFGTTVGTVEFYDPRSRNRVAVLG-----GQE---GEIT
FG08439.1 	GGIGVGSVNTAAIATNTHNLLAFGTSIGTVEFWDPRSKSRVAMLG-----GHD---GEVT
AN2077.1  	GGINTGAVNTGAIAEESHGLLAFGTTLGTVELWDPRAKGRAGVLLPPTQIGPDDARSEIT
          	*.*..*:**. *:* .:*.* ****::****::***::.*.. : ..:. * :.: .*:*

MG06500.1 	ALEFSPSGLHIATGSSTGMIQRYDLRRPVPLLKKDHGFGLPIKNIIHMTTSSQ------E
NCU03961.1	ALDYSRDGLSLALGTGTGIVQVFDLRNPRPLMRKDQGMGLPIKNIIHLTTPTE------E
FG08439.1 	ALDFSPTGLSIVTGSSTGMMQIFDLRRPVPLMKKDQGYGFAVQKLIHMTTASQ------E
AN2077.1  	ALEFHRSGLTLATGSSNGLIHLYDLRSPVPLLKKDQGYGFPIHTLKFLLPSTTTREQTLE
          	**::   ** :. *:..*::: :*** * **::**:* *:.::.: .: ..: : ..: *

MG06500.1 	KK0ILSADKKSIKIWDESSGNPWTTIEPVVDINHVAWCKGTGMLFSANEGPQMHAWLIPH
NCU03961.1	KK~LLTADKRIIKLWDEQSGDLWTSIEPTVDINHVAHVPDSGMLLTANEGKQMHSFFIPN
FG08439.1 	KK~ILSADKRIIKLWDEQTGDPWTSVEPIVDINDVAWCKDTGMIMTANEGKQQHAFFVPQ
AN2077.1  	PK~ILSSDKKIIKIWDPRNGNPWTSVEPAVDINSVAWCKDSGMLLTANEGRQQHSFFIPQ
          	 * :*::**: **:**  .*: **::** **** **   .:**:::**** * *::::*:

MG06500.1 	LGNSPRWCSFLDNLVEEMAEEVH----------TETYDDYKFLTLPELKQLSLSHLIGKS
NCU03961.1	LGLAPRWCHFLDNMVHEMENEKR----------VDTYDNYKFLTIPELKQLSLAHLVGKT
FG08439.1 	LGPAPKWCAFLDNMVEEMAEEVR----------TETYDNYKFLTLPELKQLSLSHLVGKT
AN2077.1  	LGPAPKWCSFLDNLVEEMAEDPNDPNAFNAGQAGSVYDNYKFLTLPQLKTLSLDHLIGRT
          	** :*:** ****:*.** :: ....: .:..: ..**:*****:*:** *** **:*::

MG06500.1 	NLLRPYMHGYFVAAKLYDQARLIANPYMYEEERMKRVKEKVEKERASRIRGNKK--VKVN
NCU03961.1	NLLRPYMHGYFVHSKLYDQARLIANPYVWEEERAKRVKEKVEKARESRIRGVKK--VKVN
FG08439.1 	NLLRPYMHGYFVASKLYEQARLIANPYVWEEERTKRIKDKVEKERASRIRGTKK--VKVN
AN2077.1  	NLLRPYMHGYFVAQRLYEEARLITNPYIWEEERAKRIKEKIDKERESRIRGKKKAAVKVN
          	************  :**::****:***::**** **:*:*::* * ***** **::****

MG06500.1 	QKLVDKLLKKQEKREK-----------------------VDTEAGVLGDDRFKSLFEDDD
NCU03961.1	QKLVDKIVDRSDKKK--------------------------IAAGVLGDSRFGKLFEDEE
FG08439.1 	QKLVDKLLKKQENREE-----------------------VDTKAGVLGDDRFNKMFEDEA
AN2077.1  	KKLAERLMQMEEKNERRKAKRVLQQGGDEPMEDADATASAPTPTAVLSDNRFAKVFEDED
          	:**.::::. .::.:  .:.   ....... ..:.::::    :.**.*.** .:***: 

MG06500.1 	FVIDETSREFKALNPSTVVAPQ-------------EAEIAN-----KGRSD-EDTSDDDD
NCU03961.1	FKVDETSREFRALNPSTQVGGQ-------------DVAVQHVKDDSSVTSNSDDESDPES
FG08439.1 	FQVDENTGEFRALNPSTMVEQA-------------TQPKGPTRYQAKDSDD-DQSSDEDM
AN2077.1  	FAIDETSREYSLRNSGSASEPAPPPRKERGLTAVEQEAIDEVPNSSDSDSDSESESESEQ
          	* :**.: *:   *..:     ... .. . :: .         .:.  .:::. *: : 

MG06500.1 	DEIRG--------SSK-----DGVQMRVSSSANHGKRAKDTVLGSRMPKSR-VEKTRYGG
NCU03961.1	EVESK--------KKKSKKSKDDVVMQVSSSKIAGGKAKDTAFGQRAPRTG-RVTKTRT-
FG08439.1 	DKPVA--------APKKKTKDEDVVMRVSSTSNQGRAVRDTALGSRQQKFG-RVNKAHG-
AN2077.1  	ERPSKRKPSAQKQKPANNITRRQPQMQVSSSAAAATSNRDRSFGARAQNMRTKEKPARRV
          	:     ..::...   .. .     *:***:   .   :*  :* *  .  :  .     

MG06500.1 	PGVGEKEITFVPESK--KKKKEE----PVAIRPKPS-NARRSASSNTMRKL
NCU03961.1	DVVGEQKITFVPESK--KKKQAE----PEAAPTRKRHDGRRSASTNTFRRL
FG08439.1 	EVVGERQVSFIPESR--RKQKEREDELPAEPRDKRTGAARRSASGNTFRRI
AN2077.1  	TVVGEQEFTFNPRGKGSSKQNNNAPVKPTTASSDYRTKERRSASGNTFRKM
          	  ***::.:* *..:.: *:: .    *           ***** **:*::
```
